# Beating heart mitral valve repair for a patient with previous coronary bypass: a case report and review of the literature

**DOI:** 10.1186/1749-8090-8-187

**Published:** 2013-08-30

**Authors:** Teruya Nakamura, Hironori Izutani, Naosumi Sekiya, Taro Nakazato, Yoshiki Sawa

**Affiliations:** 1 Division of Cardiovascular Surgery, National Hospital Organization Kure Medical Center/Chugoku Cancer Center, 3-1 Aoyama-Cho, Kure 737-0023, Japan; 2 Department of Surgery, Ehime University Graduate School of Medicine, Toon, Japan; 3 Department of Surgery, Osaka University Graduate School of Medicine, Suita, Japan

**Keywords:** Mitral valve insufficiency, Mitral valve repair, Reoperation, Minimally invasive surgical procedures

## Abstract

Mitral valve reoperation, through a median sternotomy, for a patient with patent coronary bypass grafts is technically challenging and carries higher postoperative morbidity and mortality than a primary operation. We present a case of mitral valve repair using a beating heart technique under normothermic cardiopulmonary bypass that was performed 3 years after a coronary artery bypass operation. A limited (10 cm) right thoracotomy was made and cardiopulmonary bypass was conducted using the ascending aortic and femoral venous cannulation. The left atrium was opened while beating was maintained. Triangular resection of the prolapsed portion of the posterior leaflet and ring annuloplasty were performed. Completeness of the repair was verified by direct visualization under beating condition and transesophageal echocardiogram. This technique is a safe and feasible option for a mitral valve reoperation that excludes re-sternotomy, extensive pericardial dissection and aortic clamping, thereby minimizes risks of bleeding, graft injury and myocardial damage.

## Background

The number of patients undergoing cardiac reoperations continues to increase. Redo procedures usually involve sternal reentry, which has the potential for hazardous injuries to the important structures and subsequent morbidity and mortality [1]. In the case of a patient who has poor ventricular function for a long-standing valve disease, cardiac arrest may predispose the dilated myocardium to ischemia-reperfusion injury and postoperative low cardiac output [2]. We describe a case in which we performed a redo mitral valve repair after coronary bypass surgery using a beating heart approach via a limited right thoracotomy. Advantages and disadvantages of the procedure are discussed.

## Case presentation

**Figure 1:**
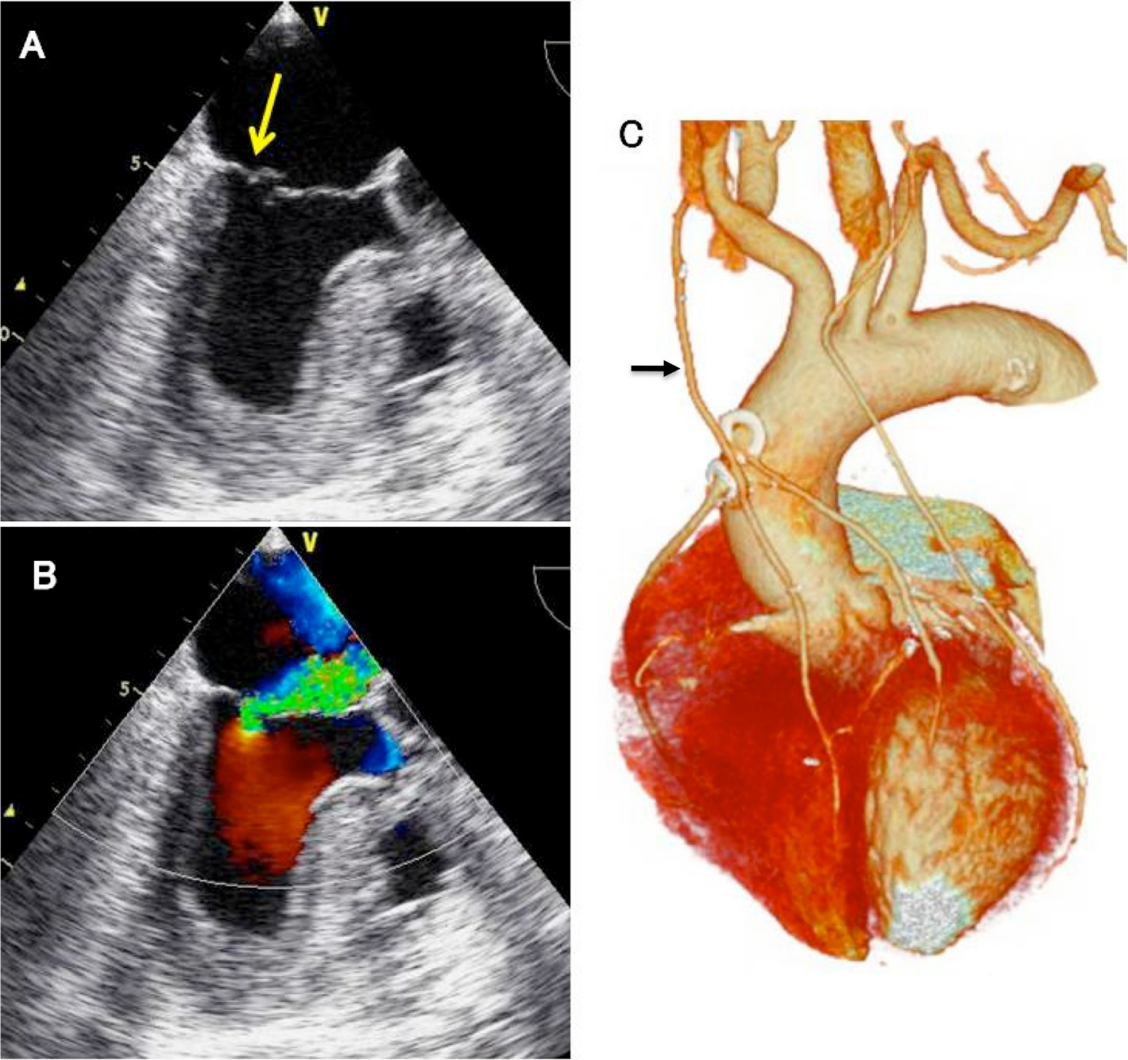
**Preoperative images of the presented case. (A)** Echocardiographic image showing prolapse of the posterior leaflet of the mitral valve (arrow). **(B)** Color Doppler image showing severe mitral regurgitation. **(C)** Computed tomographic scan showing the patent bypass grafts. Note that the right internal mammary artery (arrow) runs across the ascending aorta.

A 76-year-old Japanese male who underwent quadruple coronary artery bypass surgery 3 years before had developed shortness of breath, and visited our clinic for checkups. His postoperative course was significant for mediastinal wound infection, which was treated by local debridement and antibiotics. Upon examination, a loud systolic murmur was noted at the apical area. Chest roentgenogram showed mild cardiomegaly. Electrocardiogram showed sinus rhythm and no evidence of myocardial ischemia. Echocardiogram demonstrated severe mitral regurgitation due to torn chorda of the posterior leaflet (P2), which was not noted at the initial operation (Figure 1A, B). Otherwise, severe tricuspid and mild to moderate aortic insufficiency were noted. Cardiac computed tomography demonstrated that all bypass grafts were patent. The right internal mammary artery ran across the front of the heart and was connected to the left anterior descending branch (Figure 1C). Written informed consent was obtained from the patient and his family, and the patient was taken to the operating room for a corrective surgery.

**Figure 2:**
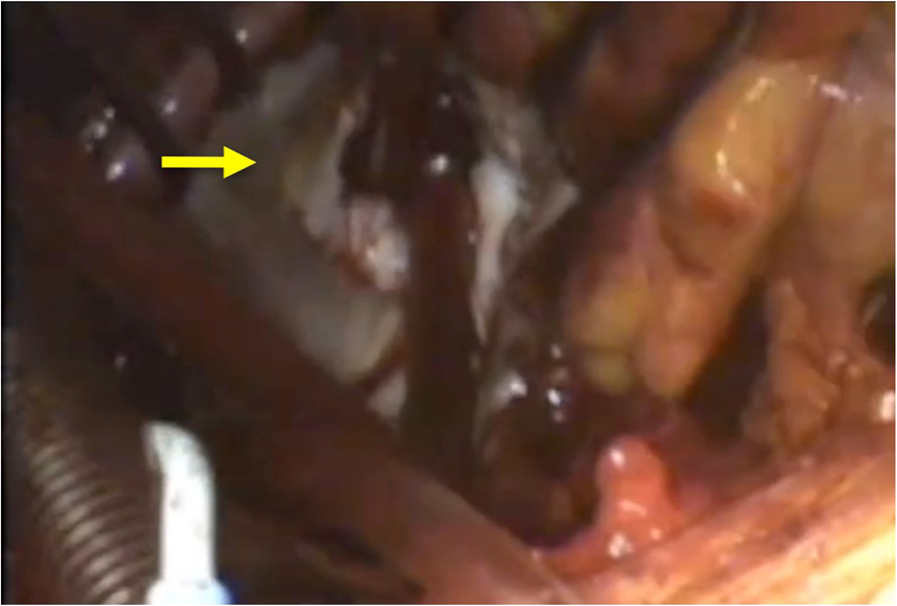
Intraoperative view of the mitral valve (arrow).

The patient was intubated with a double-lumen endotracheal tube. The patient was placed in a left hemi-decubitus position. Heart rate was maintained around 40 beats/minute throughout the procedure by remifentanyl and a short-acting beta-blocker (Landiolol) infusion. A 10 cm skin incision was made and the right lateral thoracotomy was performed via the 4th intercostals space. The lateral aspect of the ascending aorta was easily dissected and was cannulated. In order to avoid injury to the vein grafts, care was taken not to dissect the anterior aspect of the ascending aorta. The right femoral vein was cannulated with a 25 Fr. venous cannula (QuickDraw™, Edwards Lifesciences, Irvine, CA), and cardiopulmonary bypass was initiated. The superior vena cava was cannulated for additional venous drainage. Vacuum-assisted venous drainage was used to establish a full flow cardiopulmonary bypass. Carbon dioxide gas was insufflated into the pleural cavity. An aortic root vent needle was inserted. It was ensured that the aortic valve was not open by transesophageal echocardiogram. The patient was placed in the Trendelenburg position, and the left atrium was opened. Two drop-in suckers were placed, one in the left atrium and the other in the left ventricle to maintain a bloodless operative field. The mitral valve was exposed using a handheld retractor, and P2 prolapse due to torn chordae was evident (Figure 2). A standard triangular resection and suture technique was performed. A 26-mm Cosgrove-Edwards band (Edwards Lifesciences) was secured onto the posterior annulus for annuloplasty. The valve was verified competent under physiologic condition and a saline injection test was not necessary. The left atrium was closed using a standard technique. The tricuspid annuloplasty was performed using a 26 mm MC3 ring (Edwards Lifesciences). After de-airing, competency of the valve was checked by transesophageal echo (Additional file 1). The operation time and cardiopulmonary bypass time were 300 minutes and 163 minutes, respectively. Intraoperative blood loss was 320 ml, and no transfusion was required. The postoperative course was uneventful and the patient discharged on postoperative day 10. After 6 months, the patient remains asymptomatic with no evidence of recurrence of mitral regurgitation by echocardiogram.

### Discussion

As techniques in cardiac surgery continue to improve and patients who have undergone surgeries live longer, the number of redo cardiac operation increases. Redo cardiac operations can be challenging because of an increasing rate of postoperative morbidity and mortality, which is particularly true in patients with previous coronary bypass [3]. Techniques of mitral valve reoperation are becoming important because many patients have received mitral valve repair or bioprosthetic valve replacement over the last few decades, and these patients are prone to revisional operations down the road. In the present case, the patient had patent internal mammary grafts and a history of mediastinal wound infection. Intra-pericardial adhesion was expected to be dense, and it appeared quite difficult to re-enter the midline and dissect the internal mammary artery grafts. Therefore, the beating heart operation via a limited thoracotomy was a useful option as it avoided extensive dissection and injury to important structures [3,4].

There are other options for a redo mitral repair: 1) sternotomy and cardioplegic arrest or 2) hypothermic fibrillatory arrest. Of these options, beating heart mitral valve repair via limited thoracotomy offers a number of advantages. First, continuous myocardial perfusion mitigates myocardial damage caused by ischemia-reperfusion injury [5]. Although ventricular fibrillation is an alternative approach to avoid aortic clamping, it is known to reduce oxygen delivery to the subendocardium and result in suboptimal myocardial protection [2,3]. Therefore, this approach would be helpful in patients with previous bypass surgeries who had dilated ventricles and functional mitral regurgitation due to progression of ischemic cardiomyopathy [2]. As normothermic perfusion is maintained, risk of coagulopathy is reduced and blood loss is usually much less than with hypothermic fibrillatory arrest [3]. By avoiding sternal reentry, risk of excessive blood loss and wound infection are minimized. Because pericardial dissection is limited to the left atrial incision, this approach is quite safe and timesaving. As is shown in the present case, physiological assessment of repair is easily made while beating is maintained [6]. Finally, this approach can avoid systemic embolization caused by aortic clamping.

A major drawback of the approach is technical difficulties, especially in valve repair, while the heart is kept perfused and beating. Botta and colleagues reported 18 redo mitral valve cases with a similar approach; only 1 of 18 cases completed a valve repair; the remaining cases underwent valve replacement [7]. To minimize the risk of compromising the quality of valve repair, it is quite important to maintain a relatively bloodless operative field, especially when aortic insufficiency is present. Although we used 2 drop-in suckers through the left atrial incision in this particular case, a left ventricular vent via the apex using mini-left thoracotomy was useful in our previous experience. Remifentanil [8] and Landiolol [9] were helpful for heart rate reduction to prevent regurgitant blood flow from coming up to the operative field. Another concern is the possibility of air embolism. Ricci and colleagues reported 59 cases of multiple valve surgery with beating condition; no neurological deficit that could have been caused by air embolism occurred [10]. We believe that several points are important to preclude the possibility of air embolism. Also described elsewhere [11], a combination of vacuum-assisted venous drainage, carbon dioxide gas insufflation and aortic root venting can minimize the chance of air embolism. It is also important to keep the mitral valve incompetent to allow air bubbles to be ejected from the left ventricle. A saline injection test is never been applied because it would pressurize the ventricle. Furthermore, we routinely checked transesophageal echocardiogram before opening the left atrium and verified complete closure of the aortic valve even in the systolic phase, thereby ensuring that air is not sucked into the aorta.

## Conclusion

The beating heart mitral valve repair via a limited right thoracotomy is a valid option in terms of less blood loss and lower risk of injury to the hazardous vascular structures and the grafts compared to a sternal reentry option. Further studies are warranted to validate the potential benefits and the limitations of this technique.

## Consent

Written informed consent was obtained from the patient for publication of this Case report and any accompanying images. A copy of the written consent is available for review by the Editor-in-Chief of this journal.

## Competing interests

In preparing the manuscript, the authors have no commercial association or sources of support that may pose competing interests.

## Authors’ contributions

TN conducted the study, and prepared all part of the manuscript. HI helped with interpreting the data and correcting the draft. NS and TN helped with correcting the data and background literature review. YS helped conceive the study, emphasized the significance of this topic, and helped proofread the manuscript. All authors read and approved the final manuscript.

## Supplementary Material

Additional file 1 A transesophageal echocardiographic motion picture demonstrating no residual mitral regurgitation. Click here for file
